# The Following Comments Were Elicited by the Perusal of an Essay, Read in Part, by Dr. C. A. Harris, of Baltimore, before the American Society of Dental Surgeons, at Their Annual Meeting, Held in Boston, July 20th, 1842

**Published:** 1843-03

**Authors:** H. H. Hayden

**Affiliations:** Dental Surgeon, of Baltimore.


### ARTICLE III.

*The following comments were elicited by the perusal of an Essay, read in part, by Dr. C. A. Harris, of Baltimore, before the American Society of Dental Surgeons, at their annual meeting, held in Boston, July 20th,* 1842. By H. H. Hayden, M. D. Dental Surgeon, of Baltimore.

Messrs. Editors:—Believing that the issuing of periodical journals of science and literature, is, in general, intended for the promotion and diffusion of knowledge by the publishing of original essays, or reprint, with the consent and approbation of the parties concerned, of entire works, with the express intention of pointing out to the reader the correctness of the author's style, and the merits of the composition and subject, on the one hand; and on the other, to expose with suitable comments, the errors of opinion, the evil tendencies of propagating or circulating misguided views and unsound theories, either in science or morals—and, believing that the American Journal and Library of Dental Science was instituted and established with the express view to that object, we have often been at a loss to account for the apparent, nay, manifest neglect, evinced in not attending to the important points so essential to the faithful and judicious fulfilment of the object of that journal.

In the first volume of that work, the writings of Mr. John Hunter, on the natural history of the teeth, were republished with notes, by Eleazar Parmly, M. D., of New York. This was as it should be—the errors of opinion on the part of Mr. Hunter, were faithfully and candidly pointed out, and those who, before, were ready to subscribe entirely to his views, are now enabled to see, in part, and correct those opinions of Mr. Hunter, which, if adhered to, are sure to lead astray, and to confirm in error. Subsequently to the republication of Mr. Hunter's work on the teeth, numerous essays, both interesting and important in a professional point of view, have, though by no means always correct, been published, and in many instances without a single comment; and moreover, several whole treatises, emanating from men of high attainments and professional standing, have been reprinted in that journal, and, with the exception of one case, sent into the world for *the amusement* of the experienced, and the edification of tyros of the profession, with their errors unnoticed—their faults uncorrected, and their views and opinions handed over to be perpetuated through time, without the semblance of a review, or a single criticism from the legitimate organs of that work. Amongst these, Mr. Koecker's "Principles of Dental Surgery," a work replete with useful hints and valuable information, intermixed with gross faults and marks of a total disregard of professional courtesies, have been published in that journal, and presented to the public with no other comments than *high* sounding praises, without the slightest exposé of those glaring faults, which ought to have been placed in bold relief, for the benefit of the young and inexperienced of the profession. For this purpose the constitution makes express provision.* In the next place, the works, or rather the writings, of Baume, on dentition, have been handed over to us in their native dress, without a word, a single criticism or comment, other than those of high sounding encomiums on the transcendant talents and abilities of the author, in searching for and pointing out, as is believed, the mysterious, or hitherto hidden cause of laborious and painful dentition, and thereby obtaining the premium offered for the best written essay on that subject.

But will it, or can it be believed, that this work ought to have been translated, and printed in the Journal of Dental Science, and ushered forth to the world by the accredited and acknowledged organs of that journal, endorsed as in every other communication contained in that work as "good," . . . . without *any comments* or criticisms, when the talented and efficient translator would shrink from the idea of subscribing to the strange anomalies contained in the writings of that author. Is this, or ought this course to be pursued and persisted in, when the principal, nay, the ostensible object of the Journal was not the imaginary prospect of gain—but a far nobler and praiseworthy one, viz. of imparting, and more widely diffusing sound and correct knowledge of every part and portion of the dental science.

Fearing that the same, or a similar course is to be pursued, by what has already appeared in the first number of the third volume of the Journal, and learning that the same subject is to be continued

* See Articles 5 and 6.

in the second number; and moreover, knowing that opinions therein contained are not in accordance with, but conflicting with those that I have long since advocated, and am still inculcating on the same subject with the present class of dental students now attending the dental college, I find myself compelled, contrary to my inclination, but in the benevolent spirit of good neighbourhood, to come forward with a fair and honourable statement of facts, so far as I am personally concerned—and to offer some remarks on the essays now being published in the Journal.

At an annual meeting of the members of the American Society of Dental Surgeons, held, in accordance with previous arrangements, in Boston, July 20th, 1842, among other arrangements previously agreed upon, the following appears on the books of the recording secretary, Dr. S. Brown. Dr. Eleazar Parmly, of New York, delivered an introductory address. After he had finished, Dr. Hayden, of Baltimore, read a lecture on the *peculiarities* of the structure, and on the effects of some of the diseases of the maxillary sinuses. At the afternoon session of the society, and agreeably to the order of proceedings, Dr. C. A. Harris, of Baltimore, commenced the reading of a paper on the "diseases of the maxillary sinus." This paper, by a resolution, was ordered to be published, in part, with the transactions of the society.

The extraordinary coincidence in the circumstance of these two papers both treating of the same subject, and that too without any previous understanding, or concert of action between the writers of either of them, together with other incidents connected with the subject, will, I trust, justify the remarks which I shall offer, or at least serve as an apology for what may be considered an unprovoked and unseasonable interference in the affair. Be that as it may, when I was about to leave home for Boston, to attend a meeting of the society of dental surgeons, thinking it possible that there might be a deficiency of subjects, or essays for the entertainment of the members; I put into my trunk two copious lectures, which I have been in the habit of delivering for several years past to the class of dental students attending our infant college. One of these, embracing new and as yet undescribed peculiarities in the structure of the maxillary sinuses, I offered to read to the society, provided it would not interfere with other essays, or papers which other members in attendance might want to read.

The proposal was accepted, and in accordance with the arrangement already agreed upon, at 12 o'clock, M., I read the lecture, for such it was in fact, and, through the kindness of Dr. J. F. Flagg, of Boston, I was favoured with a very fine head—having, for a more complete illustration of my views, the maxillary sinuses, with their internal structure completely open and exposed to view.

At this time, I was not aware of the nature of a single sentence contained in the paper or essay that my friend and colleague, Dr. H.——, was about to read; nor do I know that he had any previous knowledge of what my lecture contained. At 4 o'clock, P. M. of the same day, Dr. H—— read a *part* of his essay on the diseases of the "maxillary sinus," and upon which we shall offer a few, and we hope, acceptable remarks.

The essay read before the American Society of Dental Surgeons, by Dr. Harris, and now being published in the third volume of the "Journal and Library of Dental Science," is entitled, "Diseases of the Maxillary Sinus."

After a few introductory observations, not altogether relevant or correct, my friend, the doctor, says: "In the remarks which I am about to offer on the diseases of the maxillary sinus, it will be unnecessary to give a *full* description of this cavity, inasmuch as that *has been done* by nearly every writer on general anatomy, since the middle of the seventeenth century." We should feel greatly obliged to our friend, the doctor, if he had particularized, or named even one or more of the writers on general or particular anatomy, that has given "a full description of this cavity," "since the middle of the seventeenth century," or even until *now*, nearly the middle of the nineteenth century, for we are bold to say that no one author, whose works have been published, has *ever given* a full and accurate *physiological* view of those cavities, not even to the day, inclusive, on which the American Society of Dental Surgeons met in the principal lecture room of the Medical College, in Mason street, Boston. Nay, we are free to assert, that our very esteemed friend, the doctor, himself, has not even "winged his snipe," so as to bring him down, as we shall endeavour to prove. It is true that our friend, the doctor, has attempted to give some feint idea—some *general* outline of the form of the maxillary sinuses, by saying, "that it is of an irregular, quadrangular shape, and situated in the middle of each superior maxillary bone, &c. &c." and "that it is *lined* by the *pituitary* membrane, and communicates with the nose, &c." Pray, dear doctor, is the pituitary membrane of the maxillary sinuses the only appendage pertaining to, or lining those cavities? If a disease of the maxillary sinuses of a muco-purulent character exist, and, as you say, that most of the diseases of the maxillary sinus are occasioned by, or depend on dental irritation, pray, is the pituitary, or, in more proper terms, the mucous membrane, the first to be affected? If an inflammation is lighted up in the periosteal tissue of the alveoli and roots of the teeth, is the mucous membrane of the sinuses the *first* to be affected by the dental irritation? If so, how is the disease of the mucous membrane propagated, without, first, having extended the inflammation prevailing in the alveoli and among the teeth, *through* the lining membrane or periosteum of the maxillary sinus, which you have neglected to mention as an appendage of those cavities—sure, my friend, Dr. H——, you need not be informed, after such *long* and so many years of *experience*—such *deep* and *extensive* researches, that whenever disease in the mucous membrane is occasioned by dental irritation, or rather inflammation, the disease in the mucous tissue is *never* idiopathic but consecutive. Well, if consecutive, what is the nature and character of the disease in the mucous tissue; and, on the other hand, if idiopathic, what is the nature and character of the disease of the mucous tissue? In answer, I beg leave to say, that the disease growing out of dental irritation and inflammation, partakes of periostitis, or chronic inflammation—the other (that is idiopathic,) is, from different causes, epidermoid inflammation, involving almost exclusively, at first, the *mucous glands*, which results in aphthæ, and extensive ulceration, the same as in the mucous tissue of the mouth, fauces, &c. &c. and which, if not seasonably arrested, becomes a source of much trouble and inconvenience and sometimes of very serious consequences. The next circumstance, in order, which claims our attention, is the following—which, so far as it serves to enlarge the historical account of the discovery of the maxillary sinuses, and the diseases incident thereto, is, as far as correct, reasonable and useful.

My friend, the doctor, observes: "it was not, however, until the knowledge of anatomy had made considerable progress, that the existence of this cavity (these cavities?) was known; Casserius, an anatomist of Padua, who flourished during the latter part of the sixteenth and early part of the seventeenth centuries, is said to have been the first to discover it; (them?) but *no correct description* of it (them?) *was given* until about the middle of the latter, and to Nathl. Highmore, author of a treatise on anatomy, published in 1651, the credit of this belongs," &c.

Now with respect to the early notice of these sinuses by N. Highmore, as being the *first* to discover them, we find that they were described, before Highmore, by Eustachius and by Paaw; yet they still retain the name of antrum-highmorianum. (See Dict. des Sciences Medicales.)

But these facts we do not consider of so much importance as the following passage, viz. "But no *correct* description of it (them?) was given until about the middle of the latter, i. e. the seventeenth century." Now it seems scarcely necessary to repeat what has already been noted, viz.—that no one author, *whose works have been published*, has ever given a full and *accurate* physiological view of these cavities: nay, we think that we are safe in saying, that our friend the doctor himself, had never, during his *long* experience and *deep research*, been made acquainted with many of the peculiarities in the structure of these cavities, until the meeting of the Society of Dental Surgeons, in Boston, on the 20th of July, 1842. If to the contrary, however, it was acknowledged by a medical gentleman of high standing in Boston, previous to the meeting on the 20th of July, that he had never, before, been made acquainted with the peculiarities of these cavities, nor the cause of the frequent *recurrence* of diseases in them, after being apparently healed, or relieved from every symptom of disease. But perhaps the views and information there mentioned, although noticed and commented upon by my worthy colleague, Dr. E. Parmly, the then president in the chair, did not originate from the right source; that had some name of high antiquity, as Cartaphilus, Casserius, or Highmore, or even L. K——r, or T. B—, been mentioned as having noted them, the game would have been noticed, sought after, and pursued with a degree of zeal and enthusiasm not surpassed by that of Sir Joseph Banks when in chase of the Emperor of Morocco,* over the wall of an innocent and unsuspecting gardener, breaking down and destroying his beautiful japonicas, anemonies, &c. (See Peter Pindar's narrative of Sir J. Banks and the Emperor of Morocco.)

2dly.—"This cavity (these cavities?) are subject to some of the most formidable and dangerous forms of disease that the medical or surgical practitioner is ever called upon to treat; and yet there are few diseases incident to the human body, that have not received more attention from writers on pathology and therapeutics than these. Diseases are here met with, over which neither the surgeon nor physician can exercise any control, and their progress is only arrested with that of the life of the unfortunate sufferer."

There is much truth in these remarks, but why not with becoming frankness state the reasons that "neither the surgeon nor physician can exercise any control over them." Because of the hidden and uncertain nature of the disease in its incipient stage—its exact location, and the almost insurmountable difficulties attending an attempt at a seasonable and successful treatment. These were difficulties which Boerhaave found in the way, and which led him to observe that, apart from these obstacles, the diseases incident to the maxillary sinuses, however formidable, would present no impediment in the way of judicious and successful treatment; for the diseases of these cavities differ in no respect from those to which *every part of the body is liable and often subject.*

3dly.—My friend, the doctor, observes, (page 24, Journal of Dental Science,) "the morbid affections of the maxillary sinus (sinuses?) are, for the most part, similar to those of the nasal fossæ; there is, however, one form of disease which seems to be peculiar to this cavity, and that is mucous engorgement. Deschamps mentions two, dropsy and purulent accumulations, but," says the doctor, "the first of these, *properly* speaking, is *never met with* in this cavity, and authors who have enumerated it among the diseases that occur here, have evidently *mistaken mucous engorgement* for it.—The fluids that accumulate here are

* A butterfly of great size.

of a mucous or muco-purulent character, except when they are the result of the disorganization of some of the surrounding parts, then they are sanious."

We cannot readily acknowledge the correctness of these remarks, more especially since having made ourselves acquainted with the form and structure of these cavities and their appendages, and, moreover, with the enlightened and correct views inculcated by Jourdain, than whom, *no man,* within our knowledge, has manifested a more profound or familiar acquaintance with all the phenomena of these cavities; and yet my friend, the doctor, has not even consented to introduce his name in this particular case—why this omission, we are unable to say, unless omitted by L. K. and T. B. We, however, are not disposed thus to pass him by, or neglect so high authority.

Before we admit or deny the correctness of the doctor's conclusions respecting a dropsy of the maxillary sinuses, we must claim an indulgence in a few interrogatories, and a few physiological remarks, in order that the subject may be placed in a clear and intelligible point of view—and 1st, is it not certain that almost every cavity of the human system is occasionally subject to dropsy?—Are not all the cavities in an especial manner liable to the same disease? As the abdomen, the thorax, the brain, the heart, or rather the pericardium? &c. Why then is a dropsy of the maxillary sinus doubted, much less denied, although the facts are sustained by the best authority? It may be asserted that the structure and economy of these cavities are of that peculiar character that renders it highly improbable that a disease of this kind could originate or pass through all the stages from the incipient to that of confirmed dropsy. For we know of but two appendages or tissues appertaining to these cavities, neither of which, under any known circumstances, could be suspected of being accessory to such a disease. The one is the lining membrane or periosteum, common to every bone in the body; the other is the pituitary or mucous membrane intimately attached to, and perhaps, in fact, sustained by the periosteum; neither of these, in a normal state, can be suspected of being the source of, or having the slightest tendency to dropsy. But let us see if there are no circumstances under which a dropsy of the antrum may prevail, and to such an extent as to soften, materially, the surrounding bones, which is uniformly allowed to be a distinctive characteristic symptom of a dropsy of the antrum maxillare.—See Jourdain's works.

Mr. Garriot, in his treatise on the diseases of the mouth, and especially on the diseases of the maxillary sinuses, observes that in case of a displacement of the walls of the antrum by an accumulation of fluids in the cavity, "some have given, very malapropos, the name of hydropsy of the sinus, to the disease. The matter may have more or less of consistence, but it is always of a purulent nature: the walls of the sinus not being at all lined by a *serous membrane*, can not well occasion the pouring out of lymph sufficient to constitute a hydropsy." But, notwithstanding the opinion of Mr. Garriot and our friend, the doctor, we find that cases of a decidedly dropsical nature have occurred without the serous tissues having been suspected of even an existence in the part; but there can be no doubt that the appendages of the maxillary sinuses, though not, as yet, specifically defined, are composed, in part, of a serous tissue, and that the periosteum of the sinuses and of the teeth is, in fact, a fibro-serous membrane. This, indeed, without any positive declaration of the fact, is strongly manifested in a highly inflamed state of the periosteum of the teeth and of their alveoli; and, moreover, we have no reason to doubt the fact in every other case of the kind in every part of the body. It is acknowledged that the periosteum of bones is susceptible of a division. "It is divided by authors into two layers; the internal layer (periosteum itself) lies close to the bone, and appears furrowed as the bone is: this is one of the finest, thinnest *membranes imaginable,* and appears upon a successful injection, to be *extremely vascular*; the reason of which is that the vessels which run to the bone, play awhile upon the surface of this *membrane* before they enter into the substance of the bone. The *external* layer is of a white, glistening appearance." (See Motherby's Dict. and others on this membrane.)

Again, in corroboration,—"Have not some *morbid characters* been attributed to the fibrous membranes, which *belong* to the *serous laminæ* that are *closely* connected with them?" Again, "The serous and fibrous membranes have a manifest tendency to adhere to each other; in most cases where they are in contact they exhibit this character:" moreover, "it is to the single membrane, the assemblage of *these two laminæ, distinct* in the preceding examples, that I give the name of fibro-serous." Again, "Besides, the intimate connection of the serous and fibro-serous membranes is often essential to the functions of the part." (See Bichat's Anatomy.)

Moreover, we are told that the secretions of almost every part of the body are, when out of the circulation, susceptible of an alteration; of being changed, of becoming vitiated, and acrid, so much so as to occasion irritation, inflammation, &c., and of being *changed* into *coagulum* and *even pus,* on the surface of the different *cavities* and *membranes.* "By disease, says Mr. John Bell, (vol. iv. p. 300, Bell's Anat.) the fluids in the cavities and cellular membranes are *altered.* In dropsy, for example, the fluid of the abdomen loses the property of coagulating, on mere exposure it comes to resemble more the serum of the blood." Again—"an inflammatory action of the vessels will throw out a fluid more coagulable, and which, in a high degree of action, will form a film of coagulable lymph, or even pus, on the surface. But in a state the reverse of inflammation, such, for example, as the debility following inflammation, a *serous effusion* will be poured out, having little tendency to coagulate." If, as Mr. Bell, further remarks, (vol. ii. p. 52 and 54,) there is an increase of the halitus, or aqua pericardii in disease (of the heart,) "if it concretes into villii and cellular substances." Or is at times somewhat viscid and "immensely increased." Is it at all improbable that a dropsy of the antrum may occur by a change in the condition of those cavities, and that too, exclusively by the increased quantity and change of the halitus in those cavities? Indeed, if there were no other cause, we consider the point as well established as that there is in all and every cavity, the constant formation of halitus, which is susceptible of an altered, or morbid condition, and thereby causing an actual dropsy; of this we feel as well assured as of the existence and altered condition of the halitus, of the pericardium, or any other cavity in the body."

4thly. My friend the doctor, observes that "man is not the only animal that is subject to morbid affections of the antrum maxillare." That Dr. Wm. Cook, found that disease of a similar character prevailed among cattle, and describes an interesting case. But, though seemingly somewhat novel, it is not so much to be wondered at; for if my friend will, instead of giving a laboured written account, first examine, he will find that some of the important structural features of the maxillary sinuses, especially in the class of ruminant animals, is very like those of the human family, being in some, irregular, with *deep fossæ* with mucous tissues, &c.

5thly. "A purulent condition of the fluids of the antrum is a very common affection, but is seldom met with in persons of *good* constitution." We have seen it, however, in persons of the most robust and strong constitutions. Again—"It seems," says my friend, "to be dependent upon a bad habit of body, and *inflammation* of the pituitary membrane of the sinus, which *last,* results more frequently from dental irritation than any other cause."

We would have wished that these remarks should have come from some other source, for it betrays a remissness on the part of my friend, the doctor, in neglecting to give, in the first place, a more satisfactory physiological description of those cavities, that the reader might at one view, fully comprehend the intent and meaning of this last expression, which, seems to be clothed in some degree of ambiguity, but which may however, be owing to the obtuseness of our own perceptions. Be that as it may, we will observe that although we have offered some remarks upon an inflammation in the mucous tissue, growing out of dental irritation, we must again advert to the subject, and inquire of our friend, the doctor, whether he considers the existing inflammation in the pituitary membrane, idiopathic, or consecutive? If of the former, we should say that it *was not occasioned* by dental irritation; if on the latter, it must necessarily have involved the periosteal tissue, in which case the therapeutical indications would have required a *very different* treatment, of which my friend says but very little. The fact is, that if we wish to become *familiar* with the physiological structure, and morbid phenomena of the maxillary sinuses, if our *physical structure* is such as not to admit of an entrance, *bodily* into them, in order to obtain a perfect reconnaissance of the entire subject, we should endeavour to concentrate our intellectual faculties into a point, so small as to allow of an admittance into those sinuses, and thereby have ample room for a free and critical examination of *all* the features, and every operation that these cavities are subject to, either in health or disease. This latter course, which, with our limited faculties, is not so difficult, we not only *practice*, but would highly recommend to *every* person who feels a desire, or *intends* to be a Doctor of the diseases of the maxillary sinuses.

6thly. The next subject that invites our attention is the following, viz: "*All* purulent conditions of the secretions of the pituitary membrane of this cavity, are by some denominated abscesses. The name, however, (as is justly remarked by Mr. Thos. Bell,) *is improper*." Now as far as respects the opinion, that "*all* purulent conditions of the secretions of the pituitary membrane," of these cavities, forming, or being abscesses, we do not hesitate to deny it. "Abscesses," says our friend, the doctor, "is a different affection, and it is one that *very seldom* occurs here." It would seem in this instance, that our friend, the doctor, leans very much towards the opinion of Mr. T. Bell, who says, (page 253,) that a mucous membrane covering in a thin layer, the whole internal surface of such a cavity, should become the seat of all the consecutive steps of true abscess, is a statement bearing on the face of it, an *obvious absurdity*. "The disease is, in fact, nothing more than an *altered secretion* of the membrane,"—and this notwithstanding the *vast* amount of the most respectable evidence to the contrary.*

Whether the opinion of our friend the doctor, upon this point, be the result of a *critical* examination of the *numerous* cases that *may* have fallen under his notice, in his *long* experience and practice, or whether it is influenced, in any degree by that of Mr. Thos. Bell, (which is by no means *impossible*,) who says that the term abscess as applied to the maxillary cavities, is improper, we are unable to say with any degree of certainty. But as the opinion of Mr. Bell upon this subject, seems to be so positive, and that of our friend, the doctor, by saying that "abscess is a

* Mr. Bell, should recollect that in case of a disease of almost any kind, in the maxillary sinus, the mucous membrane is not alone the seat of disease; the periosteal tissue or lining membrane of the sinus, is from its intimate connection with the mucous tissues, necessarily involved in the disease; and when in this state, we believe, assumes, in part, the character and office of a fibro-serous tissue.

different affection, and it is one that seldom occurs here;"—seems not only to coincide with, but to corroborate the assertion of Mr. Bell—and moreover, as it is a subject of too much importance to be suffered to remain any longer in doubt and uncertainty, we feel an inclination to endeavour, at least to clear away any, or *some* of the existing doubts, that may have been created, either by foreign or indigenous writers.

To effect this satisfactorily, it would seem necessary to engage in a critical physiological and pathological disquisition of these cavities and their appendages, in order to ascertain, not only their normal condition, but what state they are severally susceptible of in an abnormal state, under any and all conditions. This course would require more labour than is compatible with our time or inclination; and since we think it may be accomplished with far less trouble and labour, and equally satisfactory, we shall dispense with it.

As it seems unnecessary to engage in any further investigation or disquisition of the peculiarities of the structure of the maxillary sinuses, it may suffice that we confine our views to the several tissues in which almost all the diseases incident to these cavities originate or are seated.

In the first place then, it seems necessary to determine what we are to understand by, or what constitutes an abscess? Fortunately for us, we have in our casket, some precious bijous, that have escaped the vortex of universal monopoly, and which we have obtained, through different channels, from abroad, and from the "*antiquarian* book store, *Cornhill*, *Boston*," from these we think some light may be brought to bear upon this subject.

First then, we assume the ground that pus is *essential* to constitute an abscess, in the common acceptation of the term.

2ly. That pus may assume, or present, two distinct characters, viz: a purulent suppuration, and a suppuration highly morbid, nay extremely putrid. (See Kesnoy on Suppuration.)

The first of these may be the result of *altered* secretions long retained in a cavity, or in the surrounding parts of slow progress; or it may be the result of suppuration consequent upon an acute or chronic inflammation, in which latter case, the pus is exceedingly putrid and offensive. *This latter we believe* to be a *very near* approximation to an abscess.

3ly. "Perhaps the most *accurate* idea we *can form* of an *abscess*, says Dr. Thompson, is that of a hollow bag, or *cavity*, formed by the exudation of coagulable lymph." (Thompson on Inflammation, p. 322.)

4ly. The question then arises, are the maxillary sinuses susceptible of, or liable to either one or both of these states or conditions? In answer, we presume that no one will deny that those secretions which depend upon the vital forces, as, in an essential manner, the mucous lymph and the halitus of the chest—the abdomen—the pericardium, &c. &c., are liable to any and every change or alteration, after being, or having been so secreted.

This we shall endeavour to prove, and in doing which we shall be as brief in our quotations as possible, so that they prove satisfactory.

In speaking of secretions, Mr. John Bell, observes: "This interstitial membrane," (the peritoneum,) "is elastic, and being cellular, to allow of motion, its *surface* is *bedewed* with *serous* exudation. This fluid is *perpetually* passing from the extremities or sides of the lymphatic arteries, or capillaries, into the cellular membrane, and upon *all the cavities* of the body." (See Bell's Anat. vol. 4. p. 299.)

Again, says Mr. Bell: "But by *disease* the *fluids* in the cavities and *cellular membranes* are *altered.* In dropsy for example the fluids in the abdomen lose the property of coagulating on mere exposure, it comes to resemble more the *serum* of the blood; this were sufficient proof that the collection is not owing merely to diminished absorption, but that there is a *change* of *action* in the *vessels* of the *peritoneum, pleura, pericardium,* &c. "An *inflammatory action* of the vessels will throw out a fluid more coagulable, and which in a high degree of action will form a film of coagulable lymph, or *even pus* upon the surface. But in a state the reverse of inflammation, a *serous* effusion will be poured out, having little tendency to coagulate." True, but it will have a tendency, from its long retention in the maxillary sinuses, and in its daily increasing morbid qualities, to form what ultimately terminates in dropsy, or, according to Jourdain, "hydropsy of the antrum."

7th. Again, Mr. Bell, in speaking of the use of the peritoneum says, "I have seen in the *high state* of *inflammation* of this membrane *pus* formed upon the surface without ulceration, and therefore probably, from the same *exhaling* or *secreting* surface; and coagulable lymph lying in flakes upon it.

The *increase* of the *serous* discharge, forms the *common ascites;* but *whenever* the *natural secretions*, or exudation from the *peritoneum is altered*, *adhesions* are apt to form." (Bell's Anat. vol. iv. p. 29)

Not only are these secretions from the peritoneum liable to alter, but the secretions from almost all the membranous tissues of the body; and not only liable to be materially altered, but to be changed into pus. And, says Professor Monroe, as it appears that the *properties* of the *lymph exhaled upon surfaces*, and into *cavities*, differ so widely in different circumstances, and as we find that *pus is often met with in such cavities*, without ulceration, is it not probable that pus itself is merely that lymph changed in its properties by passing through inflamed vessels? The cavities of the pleura, pericardium, &c. are sometimes observed to contain *considerable quantities* of *pus* without the least marks of ulceration.—(Monroe's Anat. vol. 3, page 230)

Instances of this kind have frequently been seen. Mr. Hewson, "in one patient, found three pints of pure pus in the pericardium, without any ulcer either on that membrane or on the heart. (Ibid.) In another case, the cavity of the pleura of the right side was much distended with pus. (Ibid.)

Although many other cases could be adduced to sustain our position, enough has been said, we presume, to satisfy any one that the maxillary sinuses, are, from their peculiar situation, form and structure, as liable to purulent deposites, dropsy, &c. as the abdomen, pericardium, the chest, the peritoneum, or any other *sinus* or *cavity* in the body; not only so, but that the same agents, the same materials that are employed in the *formation* of *abscesses* in other parts of the system, are but too common in these cavities, and but too frequently evinced with all the accompanying traits of virulent abscess. Of suppuration Dr. Thompson says, "But suppuration may take place in any *texture* or organ; occurring *near* to *the skin* it produces *superficial abscesses;* at a distance from this, *deep-seated abscesses.*" Page 247.

Mr. Thos. Bell seems to have founded his opinion upon the improbability of the maxillary sinuses being liable to abscesses, on the ground that the diseases as generally applied, in this case seems to involve the whole cavity, and that too, perhaps, without the accompanying symptoms of inflammation which necessarily precede suppuration; whereas a true abscess of the antrum is, in his opinion, a disease not of the cavity at large, but one that *originates* within the general cavity and is located in a particular part, and that, after passing through the common symptoms, forms a tumour within the cavity, and which suppurates as in most cases of abscesses.' If such be the opinion of Mr. Bell, and such be the opinion of our friend, the doctor, founded upon that of Bordenaave and Mr. Bell, we will endeavor to show that they both may have misconceived the true nature of the disease, and that the tumour, in both cases, although occupying but a particular *part* of the antrum was, in fact, common to that cavity. Surely neither Mr. Bell nor my friend, the doctor, need be told that an abscess may originate primarily, and be seated in the mucous membrane of the antrum, involving, more or less, the periosteum; it may arrive at its crisis, open spontaneously and discharge its contents into the antrum, and, in strictness, be as much an abscess of that cavity as the particular case mentioned by Mr. Bell and which we shall endeavour to explain. The tumour described by Mr. Bell differs in no respect from any other abscess that may form in this cavity, only that in its incipient stage it commenced *under* the *periosteum* and next to the bone; in this state it advances slowly, attended with great pain, and swelling until suppuration follow. By this time the antrum is measurably filled with a tumour. Should it open spontaneously, as it will do if not otherwise, the antrum is nearly filled with pus. The same state of things is not unfrequent in the roof of the mouth; from some cause or other inflammation is lighted up at the internal root of one of the bicuspids, or from the internal root of the anterior or second large molar teeth of the superior jaw; the swelling and inflammation tend towards the roof of the mouth, and are *seated between* the *periosteum* and *the bone*; in this state it remains until suppuration follow, and if punctured *no matter* will be found until the lancet or other instrument be *carried*

to *the bone,* and then not always, as the matter lies in a sac at the root of the tooth. Similar occurrences take place in other parts of the body, as in a felon, and also, abscesses of the inferior jaw, occasioned by a violent inflammation seated in the periosteum of one or other of the large molars. In this instance, as in almost all others, when in a high state of inflammation, the periosteum assumes the appearance and character, in fact, of a fibro-serous membrane, and in certain states and conditions, actually performs the office of both; and this we know by the swelling terminating not only in abscess but in *mele serous* and other *tumours,* &c. the latter of which no one, we presume, will venture to assert is likely to result from an inflammation only in the *simple fibrous tissue* of the *periosteum* of a tooth, or that of the alveoli.

Again,—"Ulceration of the lining membrane," says our friend, the doctor, "is an affection less frequently met with than either of the preceding. It is rarely, if ever, *idiopathic,* but seems rather to be dependent both upon some other local malady and some specific constitutional vice." It is true that this affection of the lining membrane is "less frequently met with than either of the preceding." We should have been pleased and much gratified, had our friend given us a case in point; one which he had *personally* examined, either before or after death, in order that he might be satisfied whether the case were idiopathic or consecutive. Now the former of these two states mostly depends on the following causes, viz. high inflammatory fever, small-pox, measles, &c. and a violent cold which involves all the mucous tissues of the cavities of the head, and especially the mouth, maxillary and frontal sinuses, and fauces in ulceration, all of which may be considered idiopathic; but the consecutive, as I have already remarked, is generally lighted up by *dental irritation* which involves the true periosteum or lining membrane, but which may and does occasion an abnormal condition of the mucous membrane, from their intimate connection, but not in fact ulceration. This state of things may be more advantageously studied by examining the *peculiar* condition and appearance of the *mucus glands* of the mouth, fauces, and amygdal glands when in a state of ulceration, than the maxillary sinuses possibly can be by any person while the patient is alive, and they are *precisely* alike.

The next subject, respecting the maxillary sinuses, that invites our attention is "caries of its walls." "This," says our friend, "though *always* complicated with *one* or *more other* forms of *diseased action,* seems nevertheless to be *worthy* of *separate consideration.*" This we readily admit, and cannot help wishing that our friend, the doctor, had followed up the subject with the ardour and zeal of a true sportsman; instead of which he seems to have been diverted from his course, either to amuse himself or his readers, with remarks, not altogether unconnected with the diseases of the antrum, but by no means calculated to explain, satisfactorily, the nature and cause of "caries of its walls," viz. the cavity or walls of the maxillary sinuses.

But lest I may have misapprehended my friend, in saying that he seems to have been diverted from his course, I must be permitted to quote his own remarks; when speaking of "caries of its walls," he says, "this, though *always complicated* with *one* or *more other forms of diseased action,* seems nevertheless to be worthy of *separate consideration.*" Again, "like ulceration of the lining membrane, it is an *effect* of some *one* or *more other affections.* It may (that is 'caries of the walls,' &c.) result from *accumulation* of the *secretions* of the *sinus, ulceration,* or *from tumours.*"—These marked symptoms are all connected with, or are considered as accessory to 'caries of the walls' of the maxillary sinus, and, one would suppose, quite sufficient for caries, &c. &c.—But notwithstanding this disease being "always complicated with *one* or *more other forms* of *diseased* action," &c. and moreover, notwithstanding "it is an *effect* of *some one or more* other *affections,*" not one of which is described, and further, "may result from *accumulation* of the *secretions* of the sinus, *ulceration,*" &c., my friend seems, as before, to have left his reader to take a respite, while he indulges in a seeming digression to treat of subjects not *strictly connected* with that "which I propose to *adopt*" as being "*worthy* of *separate consideration,*"—viz. the "caries of its walls," (maxillary sinus,) and to treat of "the occurrences of fungus." But, lest I may have misconceived the connection, force, and bearing, which the following may have upon the subject which my friend, the doctor, thought "worthy of *separate con-*

*sideration,*" I shall transcribe the sentence, literally, as we have it in the Journal of Dental Science, p. 37, No. 1, vol. 2.

"The occurrence of fungus, and other kinds of tumours is less frequent than any of the preceding affections, yet this cavity is not *exempt from them,* and they constitute the most *dangerous* description of *malady* that the superior maxilla is subject to." Another digression—"Although it is probable, that in *their incipient stage, they* might in *nearly every instance,* be radically removed, it is *seldom* that they are *cured* after they have attained a very large size, and implicated in their morbid action to a considerable extent the circumjacent tissues."

Now reader—before we advance any further, pray indulge us in a simple question, viz. What essential connection or important bearing is there between existing fungus, &c., in the maxillary sinus, "the most *dangerous* description of malady" that these parts are subject to, together with the probability of *their* removal and cure, and the "*caries of the walls,*" the subject which our friend saw fit to "adopt in treating" of those diseases.

Indeed we find it hard to tell, and when we take the whole account in connection with our friend's remarks upon this, (or rather these) subjects, we are almost unavoidably, as well as involuntarily placed in the situation of Mr. Whitebread, when "the Lord's anointed," George the 3d, visited his famous brewery. (See P. Pindar's descriptions &c.)

It is well and generally known, that that famous king was celebrated for the number and variety of questions which he was in the habit of propounding, upon almost any, and all occasions, and especially on such as that of visiting, with his beloved queen, the celebrated brewery of Mr. Whitebread, where scenes so appalling—so numerous—so strange—so novel—so inexplicable, were presented to view, that majesty itself could not restrain the torrent of interrogations which like an avalanche, he poured forth pell-mell, upon poor Mr. Whitebread, who, in much perplexity and confusion, turned and said, (aside) "may I be cursed, if I know which to answer first."

However—as the game is started it would betray less than the spirit of a huntsman, to abandon the chase.

We will then indulge in a few remarks upon our friend's ideas of the probability of a *cure* of *these tumours*, for the doctor speaks of them in the plural number, as will appear, as before. "Although it is probable that in *their* incipient stage, *they* might, in nearly *every* instance be *radically removed*, it is *seldom* that *they* are *cured*," &c. &c. From the knowledge we have of the maxillary sinuses, of their structure—their economy—their appendages—and the diseases to which they are liable, and from the remarks just quoted, we are involuntarily, prompted to ask how, or by what means, are those tumours to be discovered in their incipient stage, when in fact, they are not larger than a pea, or rifle-ball? How are they to be discovered—or by what symptoms can *they* be recognized?

However, as we do not find that a further examination of this part of the subject is likely to afford any uncommon degree of instruction, and of edification, we shall pass on to page 30th of the Journal of Dental Science, where we find the following:

"If all the circumstances connected with the history of the diseases under consideration could be ascertained, I think it would be found that these affections are more frequently originated by a morbid condition of the teeth, and gums and alveolar processes, than any other cause. There are no sources of irritation to which this cavity is so much and so often exposed, as that of the dental organism. It is separated from the apices of the roots of the superior molares and bicuspides only by a very thin plate of bone, and it is sometimes even penetrated by them, so that it could scarcely be otherwise, than that aggravated and protracted disease in the teeth and alveoli, which are beneath it, should exert an unhealthy influence upon it."

It will readily be admitted that these cavities are not only liable to, but often the seat of disease growing out of, or occasioned by what is termed dental irritation; nor is the inferior jaw much less susceptible of, or less frequently the seat of very serious and long protracted diseases of a similar kind, and from the *same cause*, viz. dental irritation. Indeed, when we consider that the inferior jaw has not, at least, that extent of mucous tissue which the superior has, we are safe in asserting that the inferior jaw is more subject to a high grade of inflammation, swellings, tumefactions abscesses, and tumours of *various kinds,* than the superior, or maxillary sinuses—and that too for a very plain and obvious reason, viz. when from inflammation in the inferior jaw, which tends to suppuration, the matter thus formed, from its own specific gravity naturally tends downwards, and is discharged upon the face or within the inferior border of the jaw, whereas, in the upper, the matter, by the same law, makes its way out of the gums, or through the roof of the mouth.

But in order to a correct knowledge of these facts, it is *indispensably* necessary that we first acquire, not only a correct, but an intimate physiological knowledge of each and every organ, or part, and the diseases to which each is liable; for we trust that but few will deny that each and every organ and tissue in the human body is liable to diseases *peculiar* to *itself*, and, moreover, the therapeutical indications must be in accordance with the nature and kind of disease, in order to a successful treatment. In describing the diseases of the maxillary sinuses, much stress is laid upon the *supposed* fact, that the roots, or fangs of the subjacent teeth actually perforate the floor of the antrum, and that when inflammation is lighted up in the periosteal tissue of any one or more of the contiguous teeth, the tissues of the respective sinus must necessarily be involved in the disease. Were this the fact we should readily acquiesce in the opinion, that in cases of dental inflammation of the periosteal tissue and suppuration of the nerves, the corresponding tissues of the maxillary sinus would, to a greater or less degree, be involved in the disease. But before we admit the truth of the position, as stated, to the extent to which many would lead us to believe, let us seriously inquire into, and examine, the physiological connection of the teeth and the maxillary sinuses, in order to test the truth of the allegation. In the first place, we know that the second branch of the fifth pair of nerves passes along through the superior maxilla in a *bony canal,* and from which small twigs or branches are sent off to organize each and every root of the respective teeth. Then let us suppose that the roots of one or more teeth perforate, or pass through the floor of and into the antrum—*How,* or in what *possible* way do these nervous twigs enter the teeth? Do they leave the main branch, penetrate the floor of the antrum and then descend into the roots of the teeth? Is it pretended or is it presumed, that the roots that penetrate the floor of the antrum, likewise penetrate the periosteum and mucous membrane, and that the nerves, being associated with these tissues, pass from them into the roots of the teeth; or that these nervous twigs pass along upon the floor of the antrum, *between* the membranes and bone, and so organize the teeth? The fact is, we find it somewhat difficult to determine which of these proportions bears on its face the strongest appearance of absolute absurdity. The true state and condition of these parts are, that the second branches of the fifth pair of nerves pass along *under* the floor of the maxillary sinuses, in a small bony canal in a manner similar to that of the infra-orbitary nerve, passing under or through the orbiter plate, and out upon the face. From these branches, the small twigs are thrown off to pass into the roots of the teeth, *without passing* into the antrum. To view the subject in any other light is not only unphilosophical, but, as unreasonable as improbable. That cases have occurred in which an opening or communication has been found between the antrum and some one or more roots of teeth we will not deny, nor of which do we even entertain a doubt. But this was occasioned by an abnormal state and condition of the parts, and the reasons can be easily and satisfactory explained.

Our friend, the doctor, says, that "some are of the opinion that the maxillary sinus is never penetrated by the roots of any of the teeth, except in cases where disease has destroyed the thin plate of bone which usually covers the apices of those immediately beneath it; but that this is incorrect, is proven by the fact, that both antra are often perforated by them, which would hardly be the case were the perforations the result of disease, for it rarely happens that both cavities are affected at the same time. Indeed, I have never, except in a solitary instance, known one antrum to be perforated by the roots of the teeth, where the other was not similarly penetrated, and it is by no means uncommon for the floor of this cavity to be pierced by the apices of the roots of the first and second superior molares."

Now our friend, the doctor, need not have hesitated a *moment* in saying who *one* of those "some" is that believe that the maxillary sinus is *never* penetrated by the roots of the teeth, except in cases where disease has destroyed the thin plate of bone, &c.

The fact is, *we* have frequently in our interviews, made this assertion to our friend, the doctor, and explained our reasons for denying the fact, except as already explained. But this was before our friend, the doctor, had undertaken the amazing crusade of gathering antiquated authorities, about three years ago, and from some of which he has found out, that "it is by no *means uncommon* for the *floor* of this *cavity* to be PIERCED," &c., by the roots of the teeth.

Amazing! says the fly upon a coach-wheel, "what a dust I do kick up." Without further remarks upon this part of the subject, we will observe that with us, it has been one of deep and critical research for *more* than twenty years, (and this cause we can readily explain,) and with all our examinations, and with the aid, and assistance of more than thirty antras, in our possession laid open and freely exposed to our view, not one instance is to be seen or found where there is an opening, or communication, between the roots of the teeth and the antrum, except such as have been occasioned by disease.

Whoever wishes to give a comprehensive and correct view of the maxillary sinuses, and the several diseases, mild and malignant, to which they are liable, should, in the first place, become familiar with *all* the phenomena incident to those cavities, both in health and disease. In prosecuting this investigation, he will find that, as my friend, the doctor, says, "the occurrence of fungus, and other kinds of tumours, is sometimes seated in these cavities." In attempting at any time to describe these tumours, he will probably define them as, at least, of two distinct kinds, but variously modified—that they are of two different and distinct origins—and differ essentially in their structure. Moreover, the consequences resulting from either kind, in a state of disease, upon the surrounding parts, he will describe as, in some cases, mild and unoffending; at others, awfully alarm and repulsive in their aspect. Again—after an intimate knowledge of all these phenomena has been obtained, whoever will venture to assert that "it is probable, that in their (the tumours,) incipient stage, they *might* in *nearly every instance*, be *radically*

*removed,"*—really transcends the limits, not only of probability, but of the highest attainable degree of human skill. For setting the faculty of vision and of guessing out of view, what strength of mind or acuteness of perception, can ascertain the existence of either kind of those tumours in their incipient stage, when they were, probably, not larger than a bird shot—surrounded on every side by a bony structure, that precludes all access, except at one point—at which time no symptoms of disease are manifested—no twinges of pain are experienced, until the tumour is so far developed as to meet with resistance by the surrounding walls of the sinus? who would hazard an attempt at an operation, which must necessarily and unavoidably prove hazardous, as well as painful, and perhaps, entirely abortive? Our friend, the doctor, proceeds to enlarge and to animadvert upon the different states, and conditions of the maxillary sinuses, and in a similar strain, through three entire pages, and without saying anything further upon "caries of their walls," until, if one were disposed to assent to all that is written, he would be inclined to believe that the maxillary sinuses were the veritable locum tenens, the punctum saliens, whence all the diseases of the human body emanate, and the focus, or point to which they all concentrate.

[TO BE CONTINUED.]

