# Errata

**Published:** 1843-06

**Authors:** 


*Errata in Dr. Hayden's Comments.*—We insert the following at Dr. H's request.—Page 224, the ninth line from the top, for *proportions* read *propositions*—page 225, in the fourteenth line from the top, read ("the reason of which we can easily explain")—same page in the sixteenth line, for *antras* read *antra*.

